# A Case of Congenital Deafness, Relieved by an Operation Performed by the Patient on Himself

**Published:** 1841-02

**Authors:** S. M. Knapp

**Affiliations:** Winchester, Illinois


ART. IV.—*A Case of Congenital Deafness, relieved by an operation performed by the patient on himself.* Reported for this Journal by S. M. KNAPP, Esq., of Winchester, Illinois.

SOMETIME in the month of September, 1839, I became acquainted with the subject of this paper, Mr. John Washington, a mute, directly from the Hartford, (Conn.) Asylum, where he had been prosecuting his education for nearly seven years. Finding him very intelligent and an accomplished scholar, I took the deepest interest in him from our first acquaintance, and was particular in enquiring into his history. His father, late of the city of New York, is now a resident of Troy, Missouri, and a practising physician of some eminence. Our friend left Hartford in the spring of 1839, on account of some difficulties between him and a member of the Institution, by which he incurred the displeasure of his father, and was thereby thrown upon his own resources. During his stay at the Asylum, he had made himself master of many branches of science, and also of the Greek, Latin, French, and Italian languages; besides collecting a vast amount of general knowledge, comprising an outline of the learned] professions, and much definite knowledge of the materia medica. In this exigency, cast upon the world, and yet shut up within himself, some expedient must be resorted to in order to sustain his independent position. Knowing that his misfortune would be in some measure a passport to public favor, he resolved to travel and give phrenological charts of his own making. In this he succeeded well; his sense of feeling being very acute, he was uncommonly correct. By this means he arrived at the time above named, at this place. In conversation with him upon the cause of his deafness on our first acquaintance, he referred me to several causes defined in medical works, and from them selected the case that he supposed corresponded to his own, which is described below in his own words. By experiments I satisfied myself that the eustachian tube was unobstructed, the nerve perfectly good, and that Leschevin's mode of operation would be successful in bringing him to his hearing. Indeed, so great was my faith in the experiment, that I once promised to operate myself; but on more mature reflection I shrank from the responsibility. By the various experiments that we tried his own confidence became so strong, that he resolved to operate upon himself. An account of the procedure is thus detailed by himself:

"In 1834, while travelling in the State of New York, I became acquainted with a Quaker lady who informed me, that she was once entirely deaf and mute; and from that time to the present, I have sought with avidity for all cases where the deaf have been restored to their hearing. As several successful experiments had been made in Philadelphia, some in New York and in Baltimore, I resolved, the moment I became of age, to have the operation performed. My reason for waiting till this age was the opposition I met with from my father, who would not have the responsibility rest upon himself during my minority. I was undoubtedly deaf from my birth; my mother, however, thought I could hear until ten or eleven months old, and then grew deaf; but my father was of a different opinion; upon this point my own memory does not of course serve me. I found, by examination, that among those that were born deaf, in about five cases out of nine the auditory nerve was more or less active; most of them could hear through the eustachian tube. Many could hear the ticking of a watch when placed in the mouth, or anything which emits a piercing, acute sound; but the best way to try the experiment is to set the watch running down quickly, and a jarring will be felt in the ear, and a stinging sensation will remain for a day or two on the tympanum, and a violent headache is generally the consequence. In many instances, however, the auditory nerve is paralyzed, and of course all attempts to restore the patient will be useless. Many cases of deafness arise from sickness, in which a collection of pus takes place in the internal ear, and in the eustachian tube. Mutes differ in many respects: in some the meatus auditorius externus is entirely closed. In my case the tympanum was covered by a preternatural membrane, which, in fact, is the most common obstruction. After trying a variety of experiments I became firmly convinced, that a simple operation would restore me to the inestimable blessing and privilege of hearing. Acting upon this conviction, I conversed with my friends upon the subject; but, with the exception of one, they all doubted its practicability, and advised me not to have it done. Shut out, as it were, from the world, and firmly believing as I did, that a simple operation would accomplish the object, I was at last resolved to "do or die;" and, after reading Leschevin's mode of operating upon a similar case, I provided myself with a common thumb lancet, ground down to about one-third of its original width, so as to admit of its entrance into a common silver pencil case, with the point protruding about a line and a half, as an instrument with which to perform the operation. About a week before the successful experiment, I made the attempt, but the pain was so excruciating that I was forced to desist. *Ad interim*, I injected daily oleum amygdalæ and black drop, to soften the parts; and used every precaution to keep my nervous system in a state of rest. After several intermediate attempts to "screw my courage to the sticking point," on Monday the 10th of Febrnary, 1840, I determined upon a final effort; and after taking a common half-pint tumbler about two-thirds full of Holland gin, I went into a room by myself, and after tying a bandage round my head in order to open the lobe of the ear and confine it back close to the head, so that by casting my eyes upon a looking glass obliquely I could see the opening of the meatus auditorius, I first inserted the tube, and through it the lancet; then, summoning all my energies, I plunged it in. There seemed to be a cavity between the preternatural membrane and the membrana tympani; but I had resolved to pierce the tympanum, and so pushed the lancet firmly through. I found the resistance greater than I expected. I then turned the tube around and made another, but slighter incision, directly across the first. After drawing the lancet out, I attempted to push the tube between the lips of the incision; but the pain of the operation, together with the sudden admission of air, threw me into a series of fainting fits, or, as I was afterwards told, a number of slight spasms. Retaining the tube in the ear I had a tight bandage tied over my head, and went to bed; but amid such agony that I could not sleep. During the night there was a whirling and dizziness in my head, with explosive noises, and flashes of light before my eyes, and slight discharges of blood and yellow pus through the tube. At first, all sound was blended into one distracting tumult. In the course of twenty-four hours after the operation, the ear became more accustomed to the noise, and learned to distinguish between sounds. One fact, however, at the time struck me as remarkable; that all things were capable of giving sound by being touched, and that every thing made a noise; or, to express it as I then thought, every thing, animate and inanimate, contained within itself latent sounds. After the operation I put myself under the care of William H. Wilson, M. D., whose account is given below.

"Thursday 13th. A peculiar itching, tingling sensation in the bottom of the meatus auditorius externus, and a feeling as if the upper part of the cranium was gone, is all that remains of pain, except that acute noises send a thrilling sensation through the head.

"A mute friend of mine in this town will doubtless undergo the operation, and I am satisfied it will prove equally successful; should such be the case, notice will be duly given. Subjoined are the remarks of Dr. Wilson:"

"In the month of September last, Mr. John Washington, a mute, came to Winchester. He called upon me at my office to consult me upon the probable success of an operation upon his ear, such as is described in Cooper's dictionary.

"On the 10th of February he told me his intention to operate himself. I opposed his project, but all argument was useless. He prepared himself, as he has described, and operated with success, and heard and articulated, with some distinctness, next morning. I was called to see him, and found him laboring under a great amount of symptomatic fever. By the use of small doses of antimony and Seidlitz powders I reduced the fever; and since that time he has been comfortable."

Having given these statements, which may be fully relied on, I proceed to describe the progress of the individual in learning oral language. The morning after the operation he signified to me that he could distinguish sounds, and I entered upon a series of experiments to satisfy myself of the fact. By directing him to fix his eyes upon the floor, I uttered shrill sounds in different parts of the room, which in every instance arrested his attention. But I was still uncertain whether he could define sounds. To be fully satisfied, I gave him a pen, and turning my back upon him directed him to make a stroke for each word that I should speak. I then counted ten in irregular time, yet I heard the scratch of the pen in time with every enunciation. With this every doubt vanished. I then tried to make him articulate the vowel sounds. The first three he sounded very imperfectly, but enunciated *O* with a full, manly voice. I then pronounced my own name, which he spoke with some degree of distinctness. Recollecting that I had often seen him attempt to make a noise resembling his own name, by first noticing the position of the organs of speech in other persons, I pronounced it upon his ear, and to the astonishment of all, he uttered it in a full, deep, sonorous tone, that sounded like a voice from the grave. I then retired with him to a room, and continued with him six successive days. During the first day he was feverish and somewhat delirious, and troubled also with a profuse sweating on the side of the body with the ear that was punctured. Considerable debility, for several days, was the consequence; but the tone of his nervous system is now, the eighth day, nearly restored. In the course of the second day he succeeded in enunciating all the sounds of the alphabet, and in fixing in his mind all the combinations of the vowel and consonant sounds; so that by spelling words as he saw them in his mind's eye, he was able before night to form intelligible sentences. From this time onward his progress was so rapid, that I could discover a distinct progress, from hour to hour; and by the close of the fourth day, he was able to hold a metaphysical argument, and clothe his ideas in such language as to convey every shade of thought and emotion. His voice was at first hollow, rough, and sonorous, but it daily increases in compass, and smoothness, and his general health is nearly restored.

Many curious circumstances attend this case, which it is not necessary perhaps to detail. One fact, however, is too interesting to be omitted. Before the operation, he could recollect the appearance of his countenance as seen in the mirror; but afterwards that statue-like aspect vanished, and every line of his face was invested with an ever-varying expression, which he in vain attempted to fix in his mind. His eyes acquired a new expression; his lips became thickened and chiselled; his temper has become subdued; and his whole bearing is changed into something characteristic and commanding.

*February*, 1840.

